# Radiomics based on HRCT can predict RP-ILD and mortality in anti-MDA5 + dermatomyositis patients: a multi-center retrospective study

**DOI:** 10.1186/s12931-024-02843-w

**Published:** 2024-06-20

**Authors:** Wenzhang He, Beibei Cui, Zhigang Chu, Xiaoyi Chen, Jing Liu, Xueting Pang, Xuan Huang, Hongkun Yin, Hui Lin, Liqing Peng

**Affiliations:** 1 Department of Radiology, West China Hospital, Sichuan University, 37 Guoxue Alley, Chengdu, 610000 China; 2 Department of Radiology, Chongqing General Hospital, Chongqing University, Chongqing, China; 3 Department of Rheumatology and Immunology, West China Hospital, Sichuan University, 37 Guoxue Alley, Chengdu, Sichuan 610000 China; 4 Department of Radiology, the First Affiliated Hospital of Chongqing Medical University, Chongqing, China; 5 Biomedical Big Data Center, West China School of Medicine, West China Hospital, Sichuan University, Chengdu, China; 6 Institute of Advanced Research, Infervision Medical Technology, Beijing, China

**Keywords:** Radiomics, Dermatomyositis, Computed tomography, Interstitial lung disease, Survival analysis

## Abstract

**Objectives:**

To assess the effectiveness of HRCT-based radiomics in predicting rapidly progressive interstitial lung disease (RP-ILD) and mortality in anti-MDA5 positive dermatomyositis-related interstitial lung disease (anti-MDA5 + DM-ILD).

**Methods:**

From August 2014 to March 2022, 160 patients from Institution 1 were retrospectively and consecutively enrolled and were randomly divided into the training dataset (*n* = 119) and internal validation dataset (*n* = 41), while 29 patients from Institution 2 were retrospectively and consecutively enrolled as external validation dataset. We generated four Risk-scores based on radiomics features extracted from four areas of HRCT. A nomogram was established by integrating the selected clinico-radiologic variables and the Risk-score of the most discriminative radiomics model. The RP-ILD prediction performance of the models was evaluated by using the area under the receiver operating characteristic curves, calibration curves, and decision curves. Survival analysis was conducted with Kaplan-Meier curves, Mantel-Haenszel test, and Cox regression.

**Results:**

Over a median follow-up time of 31.6 months (interquartile range: 12.9–49.1 months), 24 patients lost to follow-up and 46 patients lost their lives (27.9%, 46/165). The Risk-score based on bilateral lungs performed best, attaining AUCs of 0.869 and 0.905 in the internal and external validation datasets. The nomogram outperformed clinico-radiologic model and Risk-score with AUCs of 0.882 and 0.916 in the internal and external validation datasets. Patients were classified into low- and high-risk groups with 50:50 based on nomogram. High-risk group patients demonstrated a significantly higher risk of mortality than low-risk group patients in institution 1 (HR = 4.117) and institution 2 cohorts (HR = 7.515).

**Conclusion:**

For anti-MDA5 + DM-ILD, the nomogram, mainly based on radiomics, can predict RP-ILD and is an independent predictor of mortality.

**Supplementary Information:**

The online version contains supplementary material available at 10.1186/s12931-024-02843-w.

## Introduction

Dermatomyositis (DM) is a multifactorial autoimmune disease, characterized by distinct dermatological findings and frequently involving extracutaneous manifestations, such as skeletal myopathy and interstitial lung disease (ILD) [1]. One subtype of dermatomyositis, with anti-melanoma differentiation-associated gene 5 autoantibodies (anti-MDA5+), is associated with a higher risk of developing ILD and rapidly progressive ILD (RP-ILD) with a high mortality rate [2, 3]. RP-ILD is particularly challenging as it rapidly progresses and is often refractory to treatment, resulting in a mortality rate ranging from 33% to 59.2% within the first 6 months after diagnosis [4, 5]. The prognosis of RP-ILD might be improved with early radical treatment, such as plasma exchange and intravenous immunoglobulin [6, 7]. However, RP-ILD prediction based on medical imaging or clinical data remains uncertain [8, 9]. 

To aid clinical decision-making, researchers have explored the relationship between clinical characteristics and RP-ILD in anti-MDA5 + DM-ILD patients [5, 10, 11]. High-resolution computed tomography (HRCT) provides high spatial resolution and excellent visualization of subtle structures, allowing for accurate detection of ILD and evaluation of their types, distribution, and severity [2, 12]. In fact, manual interpretation of HRCT images is insufficient and often lacks inter-individual reproducibility. Deep learning-based radiomics, as a powerful tool to transform the biological information contained in medical images into objective, quantitative digital information, is potential to assist personalized treatment decisions in ILD [13, 14]. Machine learning algorithms have been applied to baseline HRCT to predict the progression and classification of ILD [15–17]. In the radiomics research in anti-MDA5 + DM-ILD, radiomics based on HRCT has shown promise as a prognostic tool for predicting 6-month mortality and RP-ILD [13, 18]. Li et al. found that combining HRCT-based radiomics and clinico-radiological features can effectively predict rapid progression in anti-MDA5 + DM-ILD in a cohort of 103 patients, with a best AUC of 0.812 in the test group [18]. The advancement of radiomics in the application of anti-MDA5 DM-ILD is considerable. However, none of the studies validated the RP-ILD prediction ability of HRCT-based radiomics by using external validation datasets and a relatively large dataset. And the external validation dataset is essential to determine the prediction ability of the radiomics model in the heterogeneity dataset.

Thus, this study aimed to assess the efficacy of a radiomics approach based on baseline HRCT in predicting RP-ILD and mortality in patients with anti-MDA5 + DM-ILD.

## Methods

This retrospective study involving human participants was reviewed and approved by the ethics committee of West China Hospital and the First Affiliated Hospital of Chongqing Medical University.

### Patients

A total of 189 anti-MDA5 + DM-ILD patients who underwent CT examination from August 2014 to March 2022 in West China Hospital of Sichuan University were consecutively recruited. In addition, the anti-MDA5 + DM-ILD patients with HRCT from August 2019 to May 2022 in the First Affiliated Hospital of Chongqing Medical University were consecutively collected as the external validation dataset. The diagnosis of DM was made according to the 119th ENMC or 224th ENMC classification criteria, and clinically amyopathic DM was confirmed [19–21]. ILD was confirmed by typical radiological features in chest CT [2]. RP-ILD was defined as rapid progression of dyspnea symptoms, rapid worsening of HRCT findings, or decrease in partial pressure of oxygen > 1.33 kPa (10mmHg) within 3 months [11, 22]. Each patient was diagnosed with RP-ILD within three months after CT examination.

**Fig. 1:**
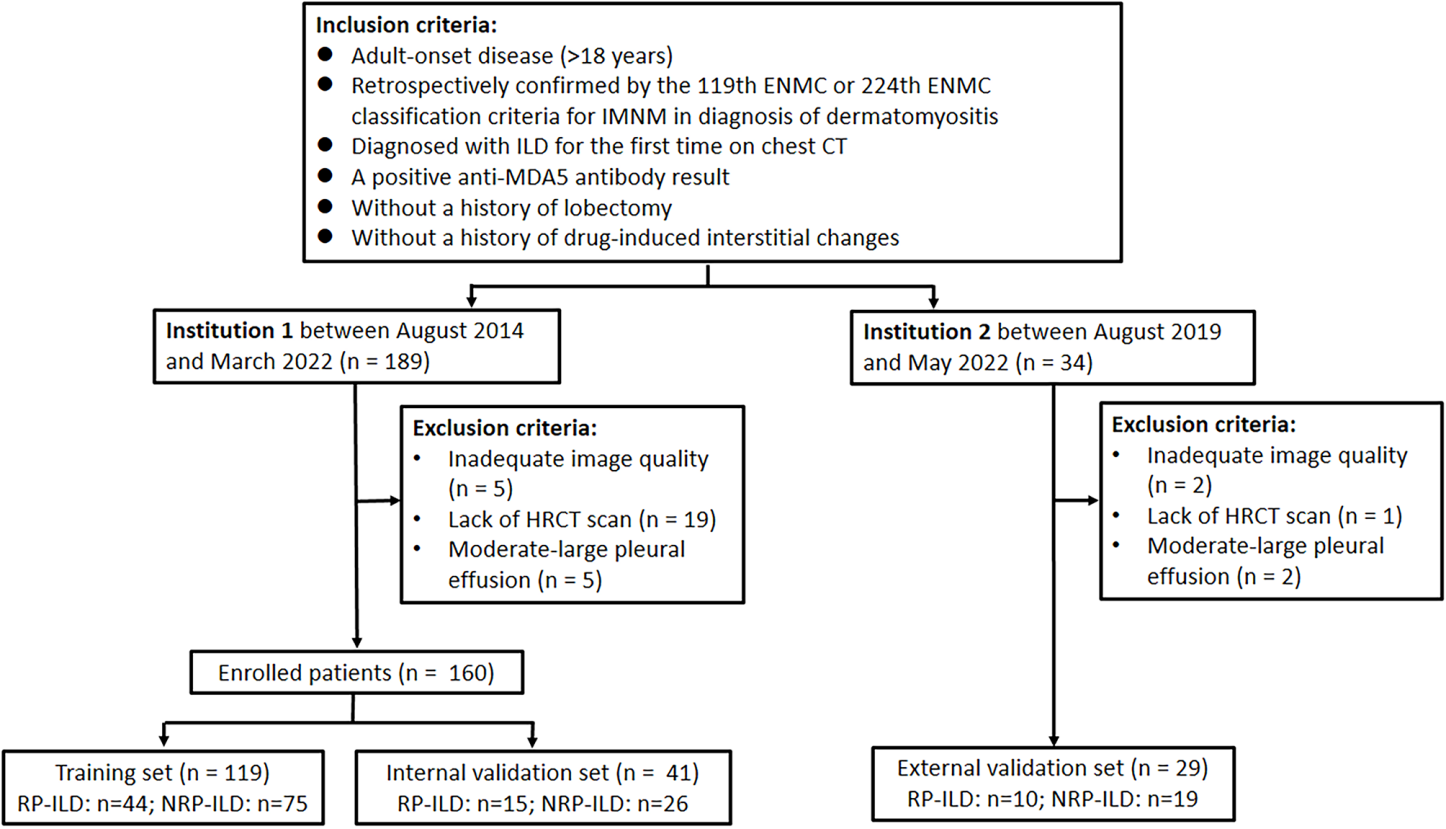
Patient enrollment flowchart. Note: HRCT, high-resolution computed tomography; RP-ILD, rapidly progressive interstitial lung disease; NRP-ILD, non-rapidly progressive interstitial lung disease

Inclusion criteria included adult-onset disease (age > 18 years), a diagnosis of DM, positive anti-MDA5 autoantibody, diagnosed with ILD for the first time on chest CT, without a history of drug-induced interstitial changes, and without a history of lobectomy. Exclusion criteria included inadequate image quality, lack of HRCT scan, and moderare-large pleural effusion. Finally, a total of 160 patients from Institution 1 were retrospectively enrolled and were randomly divided into the training dataset (*n* = 119) and internal validation dataset (*n* = 41), while 29 patients from Institution 2 were retrospectively enrolled as external validation dataset. The flowchart of patient enrollment is shown in Fig. 1. The flowchart of research is shown in Fig. S1.

### HRCT scanning parameters

HRCT scans were performed in the axial plane with 1-mm-thick sections by multidetector CT scanner including Siemens Somatom Definition (Siemens Healthcare, Erlangen, Germany). Image reconstruction convolution kernels included I70f, B10f, B30f, B80f, and B31f. In all patients, HRCT images were acquired in the supine position and at full inspiration.

### Clinico-radiologic data

All clinical data as well as laboratory samples were collected on the first day of admission and stored in the electronic medical records. Basic demographics, including age, gender, smoking history, and medical history, were assessed. The “infection” recorded in this study refers to patients with a clear diagnosis of “infection” at the time of discharge. The patients may have etiological evidence, or indirect evidence of a diagnosis of “infection”, such as symptoms, signs, and image findings suggestive of infection. Patients diagnosed with cancer on or before March 26, 2023, were documented. The clinical presentation at diagnosis, includes fever, skin changes, arthritis/arthralgia, myalgia, dyspnea, infection, oral pain/ulcers, and acataposis. Fever was defined as an armpit temperature exceeding 37.4°. Laboratory findings at diagnosis, including C-reactive protein, erythrocyte sedimentation rate, rheumatoid factor, anti-CCP antibody, creatine kinase, and blood cell count, were obtained. The neutrophil-to-lymphocyte ratio was calculated by dividing the absolute neutrophil count by the absolute lymphocyte count [11]. Myositis-specific autoantibodies and Myositis-associated autoantibodies assessments were conducted by utilizing immunoblotting technology (YHLO Biotech Co.). Positive findings in patients were validated in duplicate. Time to death was recorded as the period between the time of the HRCT examination to the time of death.

Pneumomediastinum was diagnosed based on CT images. Four HRCT-based scoring systems, namely the Idiopathic pulmonary fibrosis-score, Ground-glass opacity (GGO)-score, Consolidation-score, and Fibrosis-score, were assessed [23, 24]. The overall Idiopathic pulmonary fibrosis-score was calculated by summing the score of six zones (upper, middle, and lower zones on both sides). HRCT findings in each zone were graded 1 (normal attenuation), 2 (GGO without traction bronchiectasis), 3 (consolidation without traction bronchiectasis), 4 (GGO associated with traction bronchiectasis), 5 (consolidation associated with traction bronchiectasis), and 6 (honeycombing). GGO, consolidation, and fibrosis were separately assessed and recorded according to the pulmonary involvement area of the five pulmonary lobes. 0 (no involvement), 1 (≤ 5% involvement), 2 (5–24% involvement), 3 (25–49% involvement), 4 (50–75% involvement), and 5 (> 75% involvement) were recorded for GGO or consolidation at each lobe. And the fibrotic changes in each lobe were classified into 0 (no fibrosis), 1 (interlobular septal thickening without honeycombing), 2 (honeycombing < 25%), 3 (25–49%), 4 (50–75%), and 5 (> 75%) as fibrosis score. The respective total score of each component (GGO-score, Consolidation-score, and fibrosis-score) was the sum of each lobe’s score. Four HRCT scores were assessed independently by chest radiologists 1 and 2 (with 4 and 18 years of experience in chest imaging diagnosis, respectively) in training, internal validation, and external validation datasets. The final scores were averaged by the scores of two radiologists. One month later, 30 patients were randomly selected to be assessed by radiologist 1 to calculate the intra-observer correlation coefficient. The inter-observer correlation coefficient was calculated from the results of the first assessment by the two radiologists.

### Region of interest segmentation

**Fig. 2:**
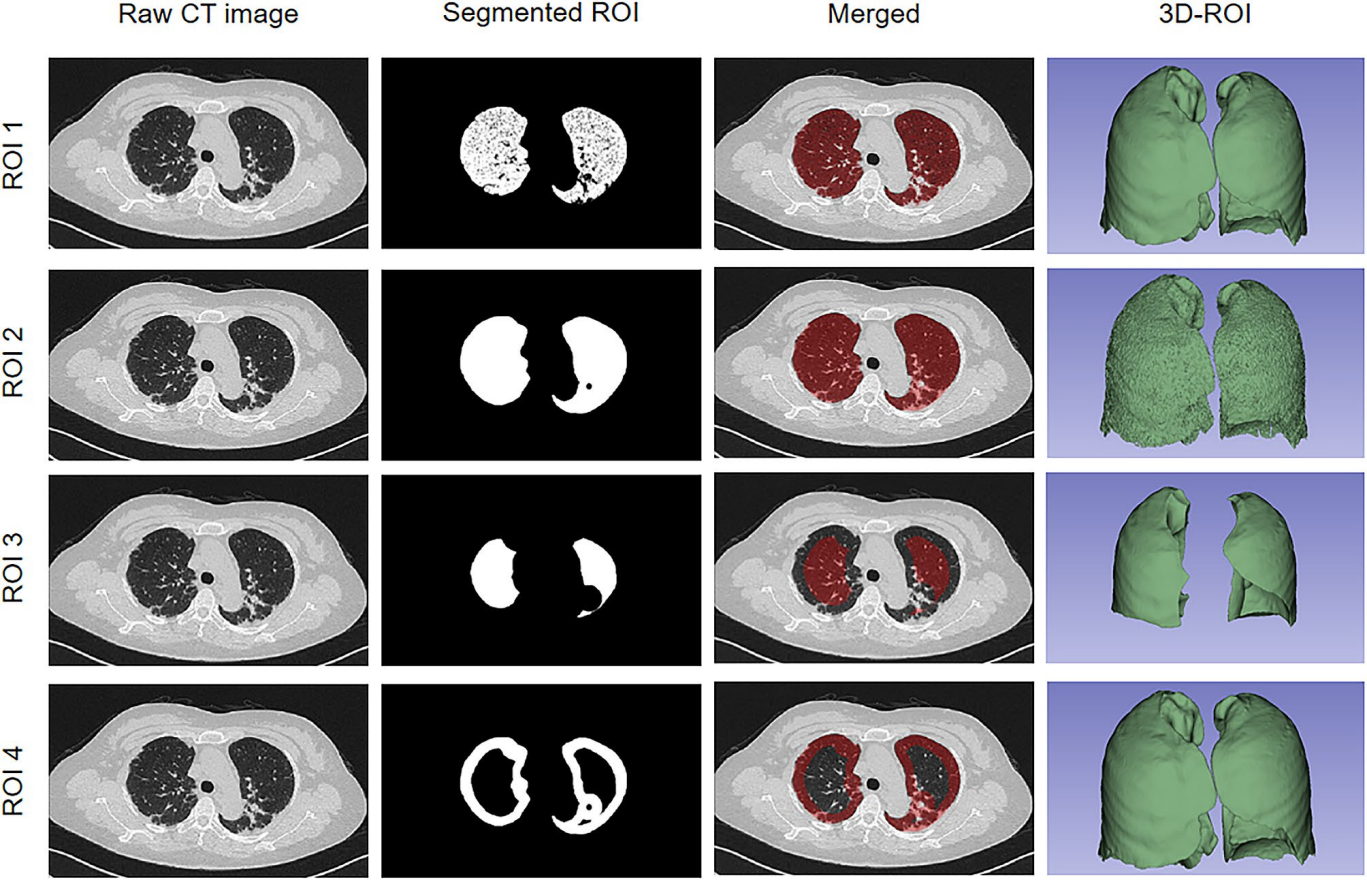
Examples of the 4 types of ROIs. Note: ROI, region of interest; 3D, three dimension; ROI 1, with Hu values from − 950 to -150 regions; ROI 2, the bilateral lung regions; ROI 3, without subpleural 1 cm area; ROI 4, the subpleural 1 cm area Note: ROC, receiver operating characteristic curve; AUC, area under the curve

The segmentation of the three-dimensional region of interest (ROI) was performed using the open-source software 3D Slicer (Version 5.0.2). The bilateral lung regions, including 5 lobes as well as corresponding bronchial and vascular bundles, were first labeled as ROI 2, while the areas only with Hu values from − 950 to -150 were labeled as ROI 1. Furthermore, the subpleural 1 cm areas were annotated as ROI 4, while the rest areas of the lung were marked as ROI 3 (Fig. 2). Three months later, 60 patients were randomly selected to be segmented by the same radiologist to calculate the intra-observer correlation coefficient. The radiologist was aware of the diagnosis of ILD but was blinded to clinical information.

### Radiomics analysis and construction of the nomogram

The pixel resampling was applied before feature extraction and the CT images were reconstructed to a target voxel of 1 mm×1 mm×1 mm. The pixel values were also converted to HU using the following formula: HU = pixel_value × slope + intercept, where slope = 1, intercept = -1024.

The radiomics features were extracted from the manually labeled ROIs in HRCT images by using the IBSI-compliant Python package named PyRadiomics (version 3.0) with the bin size fixed to 32. Multiple filters including Exponential, Gradient, Logarithm, Log-sigma (1.0 mm, 2.0 mm, 3.0 mm, 4.0 mm, and 5.0 mm), Square, Squareroot, and Wavelet (HHH, HHL, HLH, HLL, LHH, LHL, LLH, and LLL) were applied; and finally, a total of 1729 radiomics features were extracted from each ROI.

Shape features were excluded because the relationship between shape features and diffuse lung diseases is not clear. Histogram and texture features that were robust to variation in contour delineation (Intraclass correlation coefficient > 0.80) and also not highly correlated with each other (Pearson correlation coefficient > 0.95) were retained for subsequent analysis. The Least Absolute Shrinkage and Selection Operator regression analysis was applied to select radiomics features, and 10-fold cross-validation was used to select the appropriate value of the penalty parameter and avoid overfitting.

The z-score normalization was used to standardize the values of the selected histogram and texture features before model development. The formula for z-score standardization is: z-score = (X-mean)/SD, where: X represents the value of the original sample, mean represents the mean value of the original sample values, and SD represents the standard deviation of the original sample values. Four radiomics models were developed based on the selected radiomics features from ROI 1, ROI 2, ROI 3, and ROI 4, respectively. For each radiomics model, the risk-score was calculated based on the selected radiomic features with the support vector machine classifier, and the parameters were as follows: kernel = RBF, tolerance = 0.001, class _weight = balanced. In addition, the optimal c and gamma were determined by cross-validation and grid-search. By using multivariate regression, a nomogram was established by integrating the selected clinico-radiologic variables and the Risk-score of the most discriminative radiomics model in the training dataset.

### Survival analysis

The prognostic value of the nomogram was evaluated by Kaplan-Meier curves, the Mantel-Haenszel test, and Cox regression. In Institutions 1 and 2, patients were classified into high-risk and low-risk groups at a 50:50 ratio according to the score calculated by the nomogram, respectively.

### Statistical analysis

The discriminative capabilities were evaluated by the receiver operating characteristic analysis with respect to the area under the curve (AUC). Uni- and multivariable logistic regression analyses were used to select clinico-radiologic variables according to the onset of RP-ILD. The goodness-of-fit of each model was calculated via the Hosmer-Lemeshow test and the calibration curve was generated by applying the 1,000 times bootstrapping resampling method. Decision curve analysis was plotted to compare the clinical usefulness of different models.

Statistical analyses were performed on the SPSS (SPSS Institute, Inc., Chicago, IL, USA, version 26.0) and MedCalc (version 20.0) software. The Chi-square test and the analysis of variance (ANOVA) were used to compare qualitative and category characteristics. The AUCs between different models were compared by Delong’s test. The heatmap of the selected radiomics features was generated by using HemI v1.0 software. The calibration analysis and decision curve analysis were performed with R language (version 3.4.4) by using the “RMS” package and the “rmda” package, respectively. Pearson’s correlation coefficient was used to determine correlations between radiomics features, Risk-score, and four HRCT scores. A 2-tailed *p*-value < 0.05 was considered to be statistically significant.

## Results

### Patient characteristics

160 patients from Institution 1 were retrospectively enrolled and randomly divided into the training dataset (*n* = 119; 44 RP-ILD and 75 non-RP-ILD) and internal validation dataset (*n* = 41; 15 RP-ILD and 26 non-RP-ILD), while 29 patients (15 RP-ILD and 26 non-RP-ILD) from Institution 2 were retrospectively enrolled as the external validation dataset. The prevalence of RP-ILD across the three datasets was not significantly different (*p* = 0.97). Over a median follow-up of 23.6 months (interquartile range: 2.4–45.6 months), 25 patients lost to follow-up and 46 patients lost their lives (27.9%, 46/165) in Institution 1 and Institution 2 cohorts (Table S1). In our cohort, deaths occurred within six months of ILD diagnosis, and in the Institution 1 cohort (*n* = 160), there were 15 and 19 patients lost to follow-up in the first 6 months and the first 12 months, respectively. The mortality in patients with RP-ILD was higher than those without RP-ILD.

The results showed that the erythrocyte sedimentation rate and prevalence of periungual erythema were significantly higher in the external validation dataset as compared to the training dataset. However, no significant difference was observed in other clinical characteristics across the training, internal validation, and external validation datasets (all *p* > 0.05). Table 1 presents a comprehensive comparison of the demographic and clinico-radiologic characteristics of the enrolled patients in the three datasets.

**Table 1:** Demographic and clinico-radiologic characteristics of the enrolled patients

Clinical Variables	Training dataset	Internal validation dataset	External validation dataset	*p*
Female sex, n (%)	79 (66.4)	29 (70.7)	20 (68.9)	0.87
Age of onset, mean (SD), years	50.2 (11.1)	49.9 (12.7)	48.9 (11.3)	0.87
Smoking history, n (%)	19 (16.0)	9 (22.0)	2 (6.9)	0.24
RP-ILD, n (%)	44 (37.0)	15 (36.6)	10 (34.5)	0.97
Cancer, n (%)	1 (0.8)	1 (2.4)	1 (3.4)	0.53
Pneumomediastinum, n (%)	3 (2.5)	1 (2.4)	1 (3.4)	0.96
CADM, n (%)	23 (19.3)	6 (14.6)	3 (10.3)	0.46
Duration of DM, median (IQR), months	3.0 (2.0–6.0)	4.0 (2.0–7.0)	4.0 (2.0–7.0)	NA
Fever, n (%)	30 (25.2)	10 (24.4)	10 (34.5)	0.56
C-reactive protein, mean (SD), mg/L	11.3 (15.4)	12.7 (28.6)	24.6 (69.9)	0.14
ESR, mean (SD), mm/h	47.3 (21.4)	51.9 (20.5)	64.0 (27.4)	0.002
Skin changes				
Skin ulceration, n (%) Gottron papules, n (%) Heliotrope rash, n (%) Periungual erythema, n (%) Mechanic hands, n (%) Raynaud phenomenon, n (%) V sign, n (%) Shawl sign, n (%)	16 (13.4) 78 (65.5) 61 (51.3) 5 (4.1) 7 (5.9) 8 (6.7) 22 (18.5) 9 (7.6)	6 (14.6) 25 (61.0) 24 (58.5) 3 (7.3) 3 (7.3) 2 (4.9) 4 (9.8) 3 (7.3)	5 (17.2) 17 (58.6) 20 (69.0) 7 (24.1) 3 (10.3) 0 (0) 9 (31.0) 3 (10.3)	0.87 0.73 0.21 0.002 0.69 0.35 0.08 0.87
Rheumatologic manifestations				
Arthritis/arthralgia, n (%) Rheumatoid factor, n (%) Anti-CCP antibody, n (%)	76 (63.9) 13 (10.9) 6 (5.0)	26 (63.4) 4 (9.8) 1 (2.4)	16 (55.2) 3 (10.3) 3 (10.3)	0.68 0.98 0.34
Muscular manifestations				
Myalgia, n (%) Creatine kinase, mean (SD), U/L	36 (30.3) 145.6 (293.7)	12 (29.3) 126.8 (197.0)	12 (41.4) 113.7 (124.0)	0.48 0.80
Lung manifestations				
Dyspnea, n (%) Infection, n (%)	47 (39.5) 70 (58.8)	18 (43.9) 23 (56.1)	8 (27.6) 22 (75.9)	0.37 0.20
NLR, mean (SD)	5.8 (5.4)	4.8 (3.1)	6.2 (7.3)	0.43
Oral pain/ulcers, n (%)	18 (15.1)	6 (14.6)	5 (17.2)	0.87
Acataposis, n (%)	4 (3.4)	4 (9.8)	1 (3.4)	0.24

Note: RP-ILD, rapidly progression interstitial lung disease; CADM, clinically amyopathic dermatomyositis; ESR, erythrocyte sedimentation rate; CCP, cyclic citrullinated peptide; NLR, neutrophil-to-lymphocyte ratio; IQR, interquartile range.

### Selection of clinico-radiologic variables and radiomics features

The inter- and intra-individual correlation coefficients of the four HRCT scores were analyzed (Table S2). The clinical variables were analyzed by univariate and multivariate regression analysis before model development. Consolidation-score, LDH, and infection showed a *p-value* < 0.05 and were used for subsequent analysis (Table 2).

**Table 2:** Regression analysis of the clinico-radiologic variables in the training dataset

Clinico-radiologic variables	Univariate regression			Multivariate regression		
	Odd Ratio	95% CI	*p*	Odd Ratio	95% CI	*p*
**Consolidation-score**	**1.210**	**1.090–1.343**	**< 0.001**	**1.212**	**1.018–1.443**	**0.03**
GGO-score	1.146	1.062–1.237	< 0.001	1.075	0.980–1.179	0.12
IPF-score	1.151	1.056–1.254	0.001	0.914	0.761–1.099	0.34
Fibrosis-score	1.399	1.041–1.879	0.03	1.429	0.933–2.190	0.10
ALT	1.000	0.996–1.005	0.89			
AST	1.003	0.999–1.006	0.13			
CK	0.999	0.998–1.001	0.49			
CRP	1.049	1.018–1.082	0.002	1.028	0.995–1.062	0.10
ESR	1.001	0.984–1.019	0.89			
**LDH**	**1.005**	**1.002–1.008**	**< 0.001**	**1.004**	**1.001–1.007**	**0.02**
Mechanic’s hands	1.299	0.277–6.092	0.74			
NLR	1.099	1.009–1.196	0.03	1.025	0.930–1.131	0.62
Shawl sign	1.400	0.355–5.515	0.63	1.212	1.018–1.443	
V sign	0.968	0.370–2.532	0.95	1.075	0.980–1.179	
**Infection**	**4.213**	**1.781–9.969**	**0.001**	**0.914**	**0.761–1.099**	**0.03**
Age of onset	1.027	0.992–1.064	0.13			
Fever	1.430	0.616–3.326	0.41			
Arthritis/arthralgia	1.152	0.528–2.511	0.72			
Dyspnea	1.721	0.806–3.676	0.16			
Gottron papules	0.875	0.401–1.910	0.74			
Myalgia	1.124	0.502–2.517	0.78			
Periungual erythema	0.413	0.045–3.815	0.44			
Oral pain/ulcers	0.829	0.287–2.391	0.73			
Rynaud phenomenon	0.548	0.106–2.839	0.47			
Skin ulceration	0.746	0.241–2.308	0.61			
Smoking history	2.157	0.801–5.811	0.13			
Heliotrope rash	0.799	0.379–1.684	0.56			
Female sex	1.954	0.895–4.267	0.09			

Note: CI, confidence interval; ALT, alanine transaminase; AST, aspartate transaminase; CK, creatine kinase; CRP, C-reactive protein; ESR, erythrocyte sedimentation rate; LDH, lactate dehydrogenase; NLR, neutrophil-lymphocyte ratio.

There were 90 ROI 1 features, 94 ROI 2 features, 111 ROI 3 features, and 106 ROI 4 features left after intra-observer correlation coefficient analysis and Pearson correlation test. Finally, five, five, four, and four radiomics features from the ROI 1, ROI 2, ROI 3, and ROI 4 were selected for model development after LASSO regression, respectively. The optimal tuning parameter lambda was 0.10041 for ROI 1 features (Fig. S2a and Fig. S2e), 0.09402 for ROI 2 features (Fig. S2b and Fig. S2f), 0.10025 for ROI 3 features (Fig. S2c and Fig. S2g), and 0.08691 for ROI 4 features (Fig. S2d and Fig. S2h), respectively. The heatmap of these selected radiomics features according to the standardized values is presented in Fig. S3.

### Performance comparison for the radiomics models

The discriminative capability of the radiomics models was evaluated through ROC curve analysis in the training, internal validation, and external validation datasets (Fig. S4). The AUCs of the ROI 1, ROI 2, ROI 3, and ROI 4 models were 0.838, 0.898, 0.796, and 0.853 in the training dataset, 0.790, 0.869, 0.792, and 0.826 in the internal validation dataset, and 0.816, 0.905, 0.763 and 0.858 in the external validation dataset, respectively (Fig. S4a; Fig. S4b; Fig. S4c; Table 3). The detailed AUC, sensitivity, specificity, PPV, and NPV of the radiomics models under optimal threshold were summarized in Table 3. Risk-score was positively associated with IPF-score (*r* = 0.476, *p* < 0.001), GGO-score (*r* = 0.469, *p* < 0.001), Consolidation-score (*r* = 0.574, *p* < 0.001), Fibrosis-score (*r* = 0.261, *p* < 0.001) in the research cohort (*n* = 189), and more details were presented in Table S3.

**Table 3:** Performance comparison of four radiomics models

Dataset	Model	AUC	95% CI	Threshold	SEN (%)		SPE (%)	PPV (%)	NPV (%)
Training	ROI 1	0.838	0.759–0.899	> 0.2372	88.6	69.3		62.9	91.2
	ROI 2	0.898	0.830–0.946	> 0.3352	86.4	78.7		70.4	90.8
	ROI 3	0.796	0.713–0.865	> 0.3690	59.1	90.7		78.8	79.1
	ROI 4	0.853	0.776–0.911	> 0.3454	77.3	82.7		72.3	86.1
Internal validation	ROI 1	0.790	0.634–0.901	> 0.2577	73.3	80.8		68.8	84.0
	ROI 2	0.869	0.727–0.954	> 0.3371	86.7	80.8		72.2	91.3
	ROI 3	0.792	0.637–0.903	> 0.2790	93.3	57.7		56.0	93.8
	ROI 4	0.826	0.675–0.926	> 0.3234	66.7	88.5		76.9	82.1
External validation	ROI 1	0.816	0.629–0.934	> 0.3095	80.0	94.7		88.9	90.0
	ROI 2	0.905	0.738–0.982	> 0.3140	80.0	89.5		80.0	89.5
	ROI 3	0.763	0.570–0.900	> 0.3837	60.0	100.0		100.0	82.6
	ROI 4	0.858	0.678–0.959	> 0.2892	70.0	89.5		77.8	85.0

Note: AUC, area under the curve; CI, confidence interval; SEN, sensitivity; SPE, specificity; PPV, positive predictive value; NPV, negative predictive value.

### Nomogram construction

**Fig. 3:**
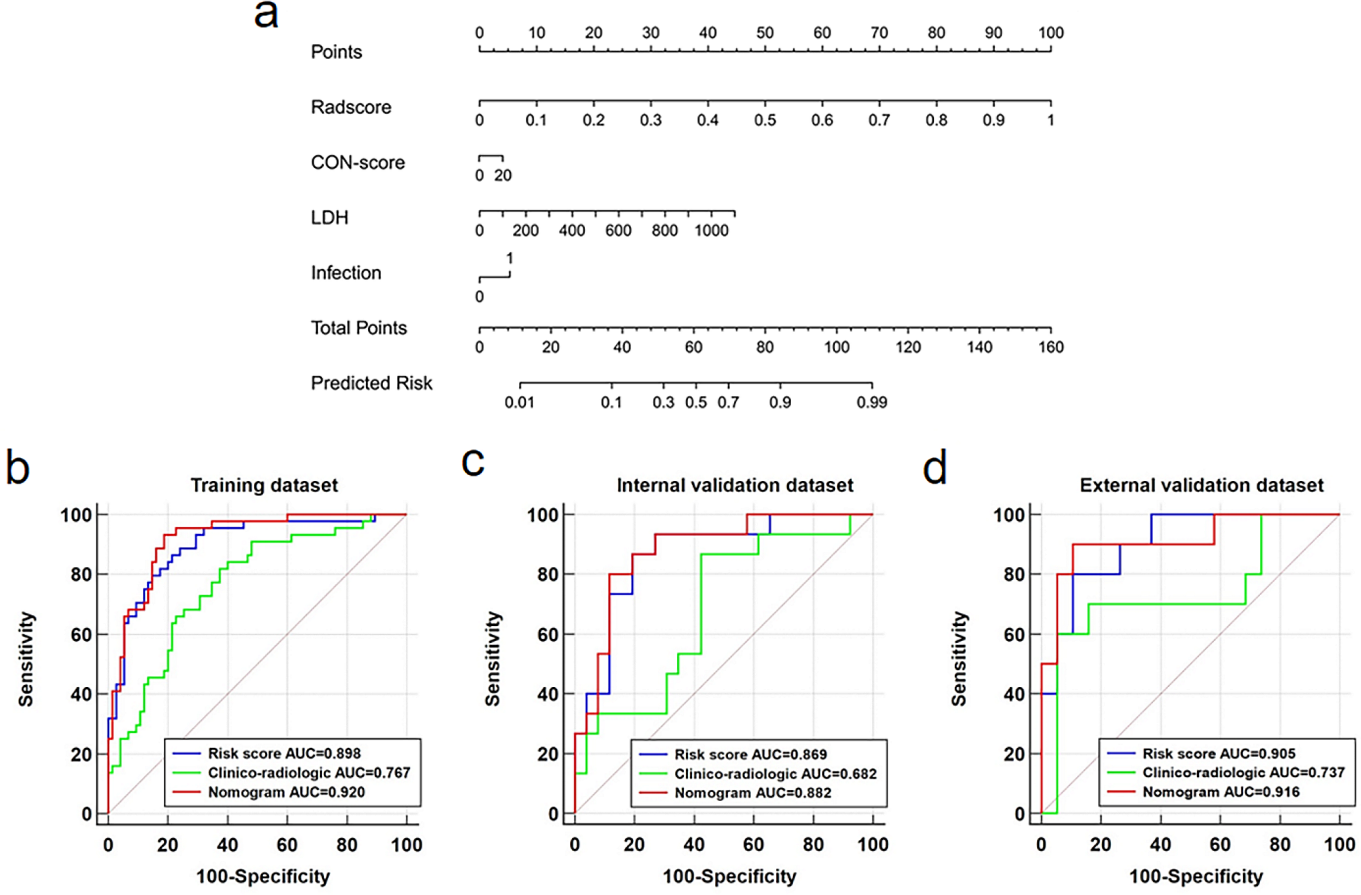
The nomogram and ROC analysis for three models. **(a)** The nomogram integrates the Risk-score and selected clinical variables; ROC analysis for the comparison of clinico-radiologic model, Risk-score, and nomogram in the training **(b)**, internal validation **(c)**, and external validation **(d)** datasets. The 45-degree dotted line represents the performance of a random classifier. Note: CON, consolidation; LDH, lactate dehydrogenase; AUC, area under the curve

By using multivariate regression, the Risk-score, Consolidation-score, LDH, and infection were used as independent predictors to build the nomogram in the training dataset (Fig. 3a). Risk-score, with a coefficient of 9.3205, was the most important variable of the nomogram and detailed information of each independent variables in the nomogram were presented in Table S4.

### Comparison of the clinico-radiologic model, Risk-score and nomogram

As shown in Fig. 3b, the AUCs of the Risk-score and the nomogram were 0.898 and 0.920 in the training dataset, which was significantly higher than that of the clinico-radiologic model (AUC = 0.767, *p* = 0.01 vs. Risk-score and *p* = 0.001 vs. nomogram). Similar performance was observed in the internal validation dataset (Fig. 3c), the Risk-score (AUC = 0.869) and nomogram (AUC = 0.882) outperformed the clinico-radiologic model (AUC = 0.682, *p* = 0.04 vs. Risk-score and *p* = 0.02 vs. nomogram). Although not statistically significant, the binary classification capability of the Risk-score (AUC = 0.905) and the nomogram (AUC = 0.916) was still higher than that of the clinico-radiologic model (AUC = 0.737, *p* = 0.13 vs. Risk-score and *p* = 0.05 vs. nomogram) in the external validation dataset (Fig. 3d). No significant differences were found between the Risk-score and the nomogram in terms of AUCs in all three datasets (all *p* values > 0.05) (Fig. 3b and c, and Fig. 3d). The detailed performance of the clinico-radiologic model, Risk-score, and nomogram was presented in Table S5.

### Clinical utility analysis

The clinico-radiologic model, Risk-score, and nomogram showed good calibration in both the internal validation dataset and the external validation dataset (Fig. S5). The non-significant statistics of the Hosmer–Lemeshow test indicated no significant deviation from an ideal fitting (Clinico-radiologic model, *p* = 0.09 and 0.14 for the internal and external validation datasets; Risk-score, *p* = 0.32 and 0.88 for the internal and external validation datasets; nomogram, *p* = 0.70 and 0.61 for the internal and external validation datasets, respectively) (Table S5).

The net benefit of the nomogram was higher than the other two models across the majority of reasonable threshold probabilities, which demonstrated that the nomogram had higher clinical usefulness (Fig. S6).

### Survival analysis

**Fig. 4:**
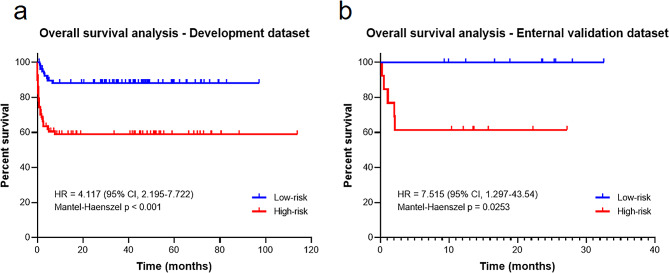
Kaplan–Meier survival analysis according to the risk score predicted by the nomogram. **(a)** Insititution 1 cohort (Development dataset); **(b)** Insititution 2 cohort (Enternal validation dataset). Note: CI, confidence interval; HR, hazard ratio

The Kaplan-Meier curves demonstrated that the high-risk group patients had significantly shorter survival time than those low-risk group patients, with hazard ratio = 4.117 (95% CI = 2.195–7.722], *p* < 0.001(Fig. 4a) in the Institution 1 cohort and 7.515 (95% CI = 1.297–43.540], *p* < 0.001 in the Institution 2 cohort, respectively (Fig. 4b).

## Discussion

The following are the main findings of our study: (1) The prediction ability of bilateral lungs, subpleural 1 cm area, -150 to -950 Hu value area, and without subpleural 1 cm area based Risk-scores ranked first, second, third, and fourth, respectively; (2) For clinico-radiologic variables, consolidation-score, lactate dehydrogenase (LDH), and infection were independent predictors of RP-ILD in anti-MDA5 + DM-ILD (OR = 1.212, 1.004, and 2.723, respectively); (3) Among the three models (Risk-score, clinico-radiologic model, and nomogram) investigated, the nomogram mainly based on Risk-score proved superior and conveyed independent biologic information for RP-ILD; (4) Survival analysis revealed that patients with higher nomogram scores had worse survival outcomes compared to those with lower scores, with hazard ratio = 4.117 (95% CI = 2.195–7.722], *p* < 0.001 in Institution 1 and 7.515 (95% CI = 1.297–43.540], *p* < 0.001 in Institution 2, respectively.

**Fig. 5:**
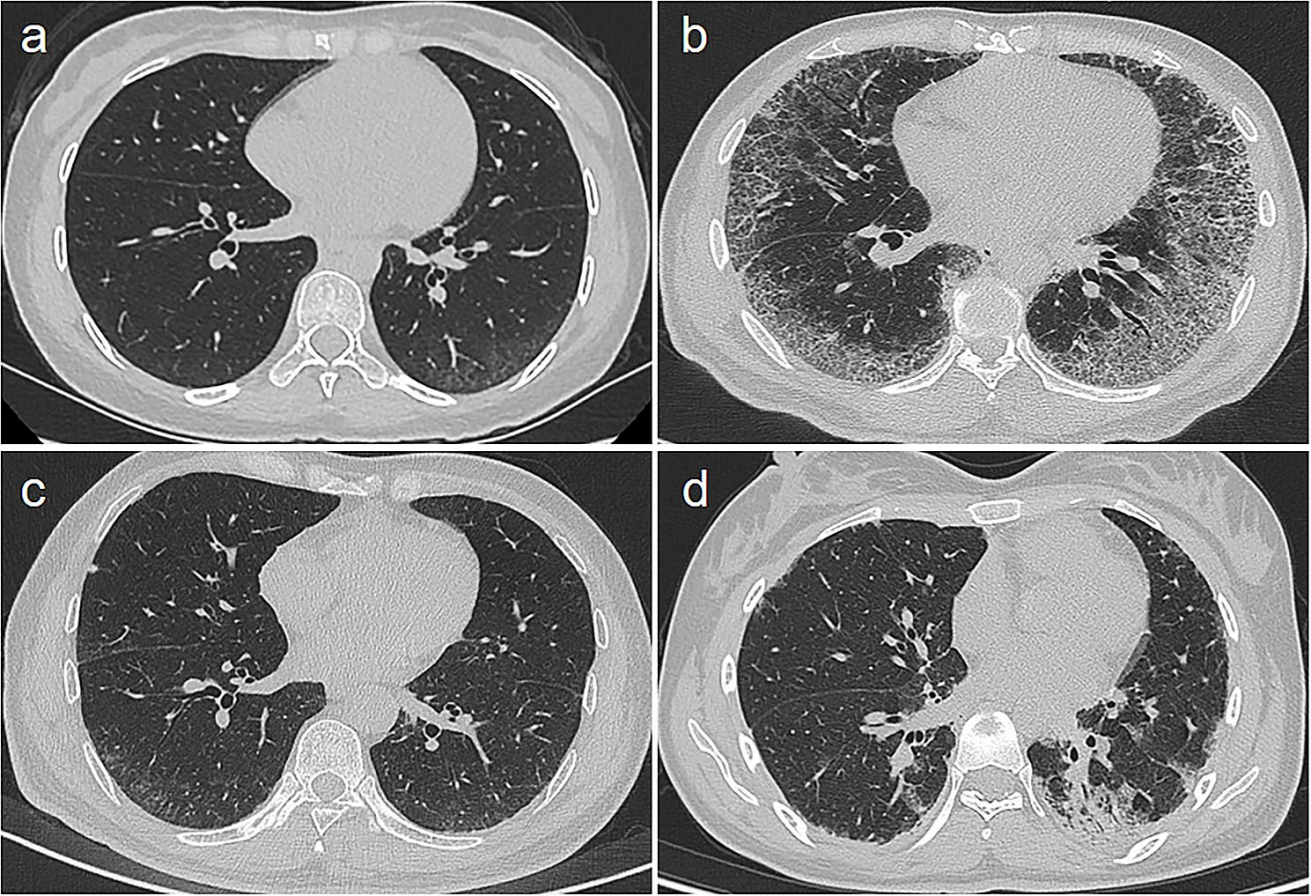
HRCT findings in anti-MDA5 + DM-ILD patients with or without RP-ILD in the external validation dataset. **(a)** A 49-year-old male patient developed RP-ILD with a Risk-score of 0.6792 and mild pulmonary findings on HRCT; **(b)** A 43-year-old male patient developed RP-ILD with a Risk-score of 0.7363 and marked interstitial pulmonary fibrosis on HRCT; **(c)** A 27-year-old female patient didn’t develop RP-ILD with a Risk-score of 0.0647 and mild pulmonary findings on HRCT; **(d)** A 22-year-old female patient didn’t develop RP-ILD, with a Risk-score of 0.1684 and significant consolidation on HRCT. The optimal cut-off value of the Risk-score for predicting RP-ILD is 0.3140. Note: HRCT, high-resolution computed tomography; RP-ILD, rapidly progressive interstitial lung disease

This was the first study to evaluate segmentation methods on radiomics analysis performance. Risk-score-based predictions might be not consistent with the severity of pulmonary signs in HRCT, and radiomics provides information beyond the radiological signs and aids in clinical decisions (Fig. 5). The radiomics analysis based on the bilateral lungs had the best efficacy in predicting RP-ILD, this might be explained by this model could mine all the potential information that could reflect the histopathological alterations in ILD patients [25, 26]. But the detailed histopathological changes on anti-MDA5 + DM-ILD are necessary to be conducted in the future. In addition, the radiomics analysis based on a subpleural 1 cm area had a fair performance for predicting RP-ILD in anti-MDA5 + DM-ILD patients, which might be explained by the most lesions located at the peripheral zones of bilateral lungs. On the contrary, the radiomics analysis based on without subpleural 1 cm area had the worst performance in predicting RP-ILD, this is easy to understand as this segmentation includes the least lesions of anti-MDA5 + DM-ILD. As for radiomics based on segmentation of -150 to -950 Hu value area, this covers most lesions that could reflect the histopathological alterations in anti-MDA5 + DM-ILD patients; however, some features such as consolidation, patchy lesions that have predicting significance have not been included.

On the other hand, radiomics analysis from different ROIs had a significant impact on feature stability and model performance [27, 28]. Incomplete ROI-based radiomics analysis may result in incomplete information capture. To avoid heterogeneity caused by different data sources, we tried to preprocess images, including resampling and discretization [29, 30]. 

In our study, the consolidation-score, LDH, and infection were found to be independent predictors of RP-ILD. Van Krugten et al. found that the lower lung zone consolidation on HRCT in anti-MDA5 + DM-ILD patients was prone to develop RP-ILD [31]. The correlation between lung consolidation and the occurrence of RP-ILD was validated in our study. Elevated LDH, associated with biological information inside and outside the lungs, reflects a severe degree of disease [32]. Infection is a common comorbidity symptom in DM patients, with pulmonary infection being the most common. Pulmonary macrophage infiltration in DM-ILD patients may be the pathogenesis of RP-ILD due to the destruction of the original physiological balance [31, 33]. 

In predicting RP-ILD in anti-MDA5 + DM, our nomogram, mainly based on Risk-score, achieved 80.0%, and 90.0% accuracies, and 88.5%, and 89.5% specificities in the internal and external validation datasets, respectively. Its calibration curve showed good agreement between predicted and actual RP-ILD, and decision curve analysis verified its clinical usefulness. The survival analysis based on the nomogram revealed that the patients in the high-risk group had significantly shorter survival times than those in the low-risk group. These findings highlighted that the nomogram might be promising in clinical practice to help predict RP-ILD in anti-MDA5 + DM-ILD patients. The study of So et al. proposed the “FLAW model”, including fever, LDH > 300 IU/l, age > 50 years, and NLR > 7 at diagnosis, which allows rapid clinical risk stratification for the imminent RP-ILD at anti-MDA5 + DM-ILD patients [11]. Li et al. identified radiomics features combined with disease duration times and dyspnea could accurately predict RP-ILD with an AUC of 0.812 in the test dataset [18]. This is partly consistent with our study, but our study did not find a potential association between disease duration time and RP-ILD as well as dyspnea and RP-ILD. In the combined model of Li et al., significant differences in AUCs in the training and the test datasets may suggest the possibility of overfitting [18]. Due to its small sample size and lack of external validation, the application of its model may require further validation. Our nomogram, combining Risk-score, LDH, Consolidation-score, and infection, would be more beneficial for predicting RP-ILD in anti-MDA5 + DM-ILD.

Our study had several limitations. First, some biological indicators as predictors or prognostic factors for RP-ILD, such as lung function test data, ferritin, and IL-1β, were incomplete. Second, a small part of patients with RP-ILD were lost to follow-up after discharge. Finally, although our cohort is already a relatively large cohort in anti-MDA5 + DM-ILD, a large sample size study from multi-centers is needed. Finally, the measurement of baseline right heart and pulmonary artery parameters on HRCT is beneficial for prognostic evaluation of lung diseases, and we will explore this in the next step of our research [34]. 

In conclusion, the nomogram developed by integrating radiomics features and clinico-radiologic factors can predict RP-ILD in patients with anti-MDA5 + DM-ILD and is an independent predictor of mortality.

### Electronic supplementary material

Below is the link to the electronic supplementary material.

Supplementary Material 1

## Data Availability

No datasets were generated or analysed during the current study.

## Abbreviations

Anti-MDA5 + DM-ILD: Interstitial lung disease in anti‑MDA5 antibodies positive dermatomyositis
HRCT: High-resolution computed tomography
AUC: The area under the receiver operating characteristic curve
Risk-score: Radiomics model
DM: Dermatomyositis
RP-ILD: Rapidly progressive ILD
ILD: Interstitial lung disease
ROI: Region of interest
LDH: Lactate dehydrogenase
