# Supplementary material for: Radiomics based on HRCT can predict RP-ILD and mortality in anti-MDA5 + dermatomyositis patients: a multi-center retrospective study
Source: Respir Res. 2024 Jun 20;25:252. doi: 10.1186/s12931-024-02843-w (PMC11191144; doi:10.1186/s12931-024-02843-w)
Supplement: Supplementary file 1 — Supplementary Material 1 [file 12931_2024_2843_MOESM1_ESM.docx]

**Radiomics based on HRCT can predict RP-ILD and mortality in anti-MDA5+ dermatomyositis patients: A multi-center retrospective study**

| **Table S1** Follow-up and all-cause mortality data. | | | | |
| --- | --- | --- | --- | --- |
| Cohort | Mortality | RP-ILD (%) | Non-RP-ILD (%) | ILD (%) |
| Institution 1 and Institution 2 cohorts (n= 189) | Overall mortality | 51.6 (33/54) | 11.7 (13/111) | 27.9 (46/165) |
|  | 3 month mortality | 52.5 (32/61) | 11.3 (13/115) | 25.6 (45/176) |
|  | 6 month mortality | 57.9 (33/57) | 11.4 (13/114) | 26.9 (46/171) |
|  | 12 month mortality | 51.6 (33/54) | 11.7 (13/111) | 27.9 (46/165) |
| Institution 1 cohort (n= 160) | Overall mortality | 63.0 (29/46) | 12.6 (12/95) | 29.1 (41/141) |
|  | 3 month mortality | 53.8 (28/52) | 12.4 (12/97) | 26.8 (40/149) |
|  | 6 month mortality | 59.2 (29/49) | 12.5 (12/96) | 27.3 (41/145) |
|  | 12 month mortality | 63.0 (29/46) | 12.6 (12/95) | 29.1 (41/141) |
| Institution 2 cohort (n= 29) | Overall mortality | 50.0 (4/8) | 6.3 (1/16) | 20.8 (5/24) |
|  | 3 month mortality | 44.4 (4/9) | 5.6 (1/18) | 18.5 (5/24) |
|  | 6 month mortality | 50.0 (4/8) | 5.6 (1/18) | 19.2 (5/26) |
|  | 12 month mortality | 50.0 (4/8) | 6.3 (1/16) | 20.8 (5/24) |
| Note: RP-ILD, Rapidly progressive interstitial lung disease; ILD, Interstitial lung disease. | | | | |

| **Table S2:** Inter- and intra-individual correlation coefficients of HRCT features**.** | | | | | |
| --- | --- | --- | --- | --- | --- |
| Variables | Inter-observer (n = 160) | |  | Intra-individual (n = 30) | |
|  | ICC (95% CI) | *p* |  | ICC (95% CI) | *p* |
| CON-score | 0.986 (0.981-0.991) | <.001 |  | 0.904 (0.805-0.954) | <.001 |
| GGO-score | 0.931 (0.906-0.950) | <.001 |  | 0.775 (0.576-0.888) | <.001 |
| Fibrosis-score | 0.986 (0.981-0.991) | <.001 |  | 0.887 (0.775-0.946) | <.001 |
| IPF-score | 0.986 (0.981-0.990) | <.001 |  | 0.852 (0.709-0.928) | <.001 |
| Note: ICC, intra- or interclass correlation coefficient; CI, confidence interval; CON, consolidation; GGO, ground glass opacity; IPF, idiopathic pulmonary fibrosis. | | | | | |

| **Table S3** Association (correlation coefficients) between bilateral lungs based radiomics features and HRCT-based scores. | | | | | | | |
| --- | --- | --- | --- | --- | --- | --- | --- |
| Cohort | Variables | Feature A | Feature B | Feature B | Feature D | Feature E | Risk-score |
| Research cohort  (n = 189) | IPF-score | -.599** | -.299** | -.340** | -.059 | .329** | .476** |
|  | GGO-score | -.589** | -.372** | -.552** | -.008 | .320** | .469** |
|  | CON-score | -.727** | -.280** | -.348** | -.149* | .323** | .574** |
|  | Fibrosis-score | -.347** | -.243** | -.186* | .061 | .183* | .261** |
| Training dataset  (n = 119) | IPF-score | -.615** | -.258** | -.355** | -.032 | .318** | .462** |
|  | GGO-score | -.607** | -.388** | -.536** | -.005 | .278** | .490** |
|  | CON-score | -.734** | -.229* | -.405** | -.149 | .313** | .569** |
|  | Fibrosis-score | -.353** | -.220* | -158 | .122 | .143 | .235* |
| Internal validation dataset  (n = 41) | IPF-score | -.655** | -.491** | -.194 | -.199 | .207 | .594** |
|  | GGO-score | -.583** | -.350* | -.571** | -.111 | .463** | .453** |
|  | CON-score | -.735** | -.424** | -.125 | -.217 | .233 | .594** |
|  | Fibrosis-score | -.554** | -.352* | -.292 | -.209 | .363* | .476** |
| External validation dataset  (n = 29) | IPF-score | -.589** | -.360 | -.657** | -.059 | .569** | .610** |
|  | GGO-score | -.542** | -.399* | -.664** | .129 | .310 | .482** |
|  | CON-score | -.685** | -.306 | -.565** | -.001 | .559** | .587** |
|  | Fibrosis-score | -.183 | -.319 | -.298 | -.068 | .251 | .288 |
| Note: *, *p*<0.05; **, *p*<0.01; Feature A, firstorder_10Percentile_log-sigma-4-0-mm-3D; Feature B, ROI2-ngtdm_Strength_exponential; Feature C, glszm_LowGrayLevelZoneEmphasis_gradient; Feature D, glszm_HighGrayLevelZoneEmphasis_log-sigma-4-0-mm-3D; Feature E, firstorder_Mean_wavelet-HHL; CON, consolidation; GGO, ground glass opacity; IPF, Idiopathic pulmonary fibrosis. | | | | | | | |

| **Table S4** Regression coefficients of the predictors in the nomogram. | | | | |
| --- | --- | --- | --- | --- |
| Variables | Coefficient | Std. Error | Wald | *p* |
| Risk-score | 9.3205 | 1.4869 | 39.295 | <0.001 |
| CON-score | -0.0035 | 0.0656 | 0.0028 | 0.96 |
| LDH | 0.0038 | 0.0017 | 5.0886 | 0.02 |
| Infection | 0.4946 | 0.4588 | 1.1622 | 0.28 |
| Note: CON, consolidation; LDH, lactate dehydrogenase. | | | | |

| **Table S5** The performance of the clinico-radiologic model, Risk-score and nomogram. | | | | | | | | |
| --- | --- | --- | --- | --- | --- | --- | --- | --- |
| Dataset | Model | AUC | 95% CI | Threshold | SEN (%) | SPE (%) | PPV (%) | NPV (%) |
| Training | Risk-score | 0.898 | 0.830-0.946 | >0.3352 | 86.4 | 78.7 | 70.4 | 90.8 |
|  | Clinico-radiologic | 0.767 | 0.680-0.839 | >0.2886 | 81.8 | 62.7 | 56.3 | 85.5 |
|  | Nomogram | 0.920 | 0.856-0.962 | >0.3116 | 93.2 | 81.3 | 74.5 | 95.3 |
| Internal validation | Risk-score | 0.869 | 0.727-0.954 | >0.3371 | 86.7 | 80.8 | 72.2 | 91.3 |
|  | Clinico-radiologic | 0.682 | 0.518-0.818 | >0.2291 | 86.7 | 57.7 | 54.2 | 88.2 |
|  | Nomogram | 0.882 | 0.743-0.962 | >0.4165 | 80.0 | 88.5 | 80.0 | 88.5 |
| External validation | Risk-score | 0.905 | 0.738-0.982 | >0.3140 | 80.0 | 89.5 | 80.0 | 89.5 |
|  | Clinico-radiologic | 0.737 | 0.541-0.882 | >0.5047 | 60.0 | 94.7 | 85.7 | 81.8 |
|  | Nomogram | 0.916 | 0.752-0.986 | >0.2754 | 90.0 | 89.5 | 81.8 | 94.4 |
| Note: AUC, area under the curve; CI, confidence interval; SEN, sensitivity; SPE, specificity; PPV, positive predictive value; NPV, negative predictive value. | | | | | | | | |

**
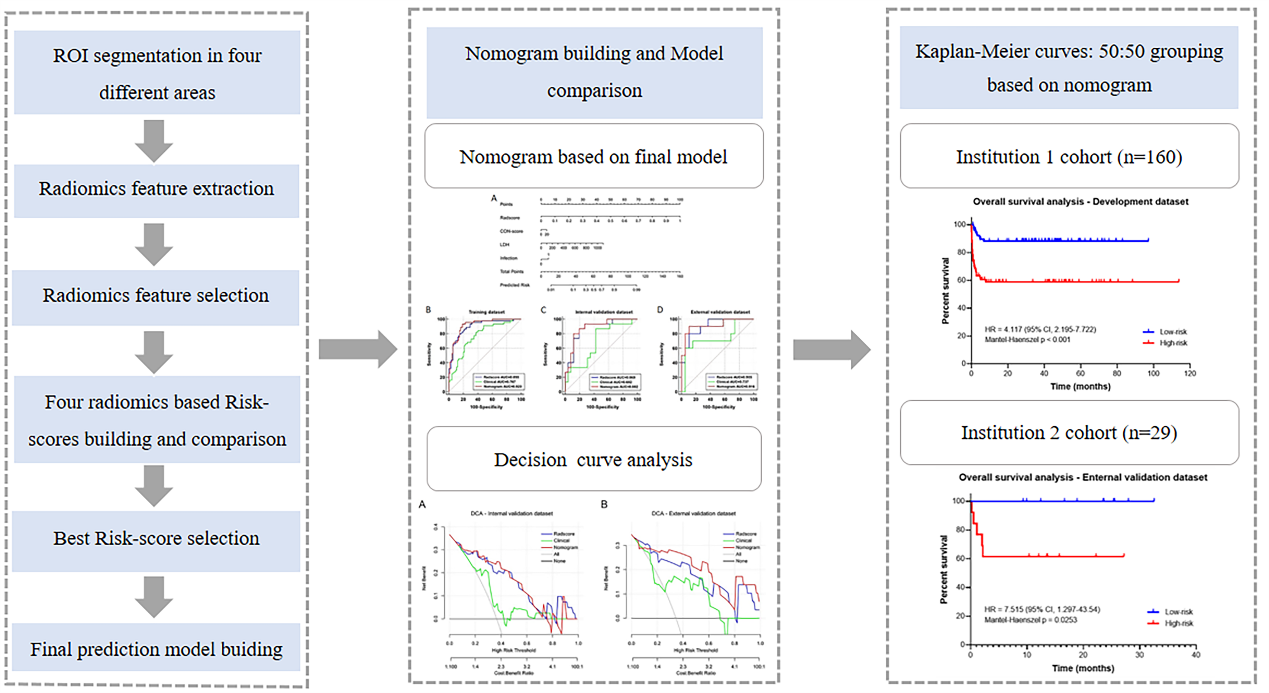
**

**Fig. S1** Research flowchart.


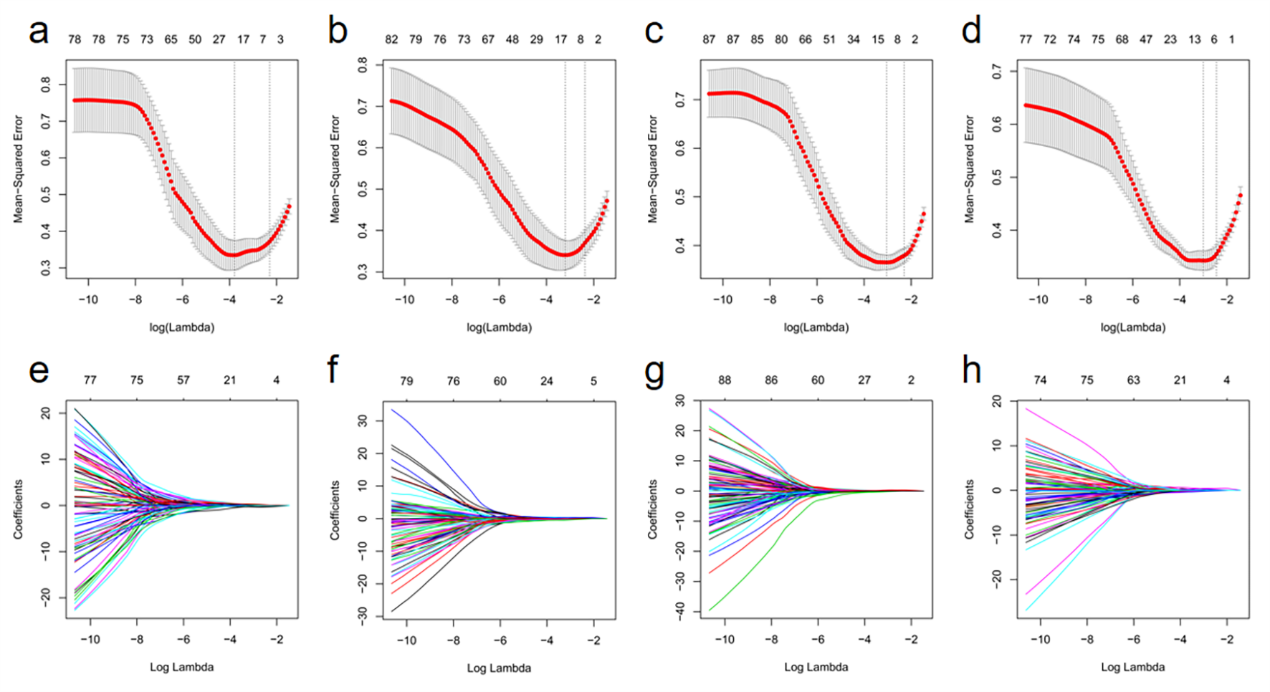


**Fig. S2** LASSO regression.


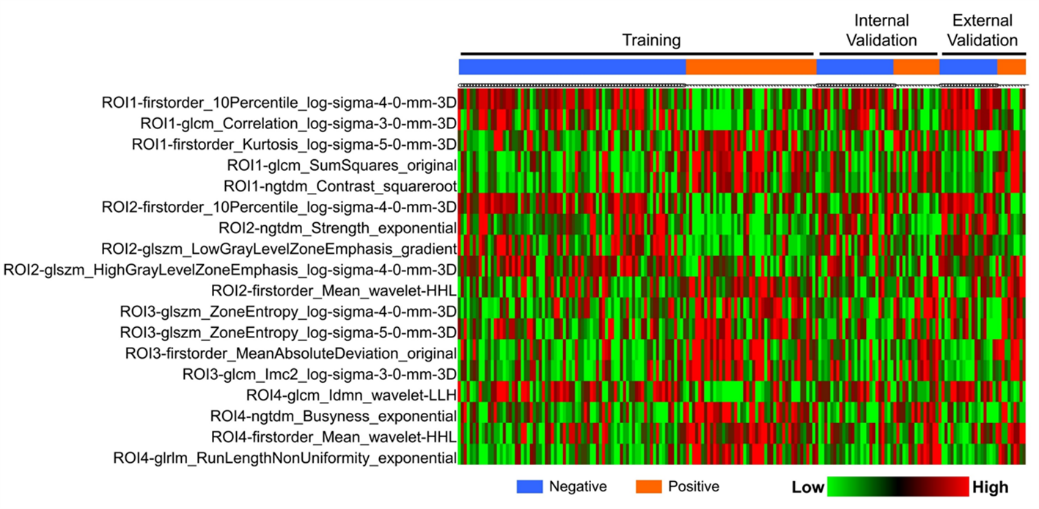


**Fig. S3** Heatmap of the selected radiomics features.


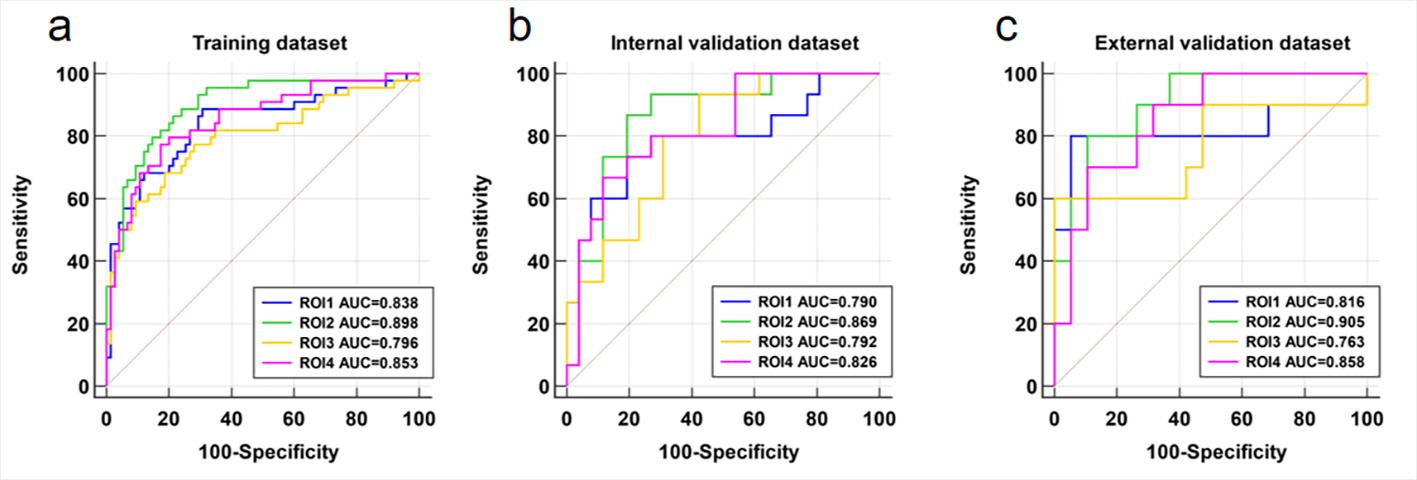


**Fig. S4** ROC analysis of the radiomics models in the training dataset (a), internal validation dataset (b), and external validation dataset (c), respectively. The 45-degree dotted line represents the performance of a random classifier.


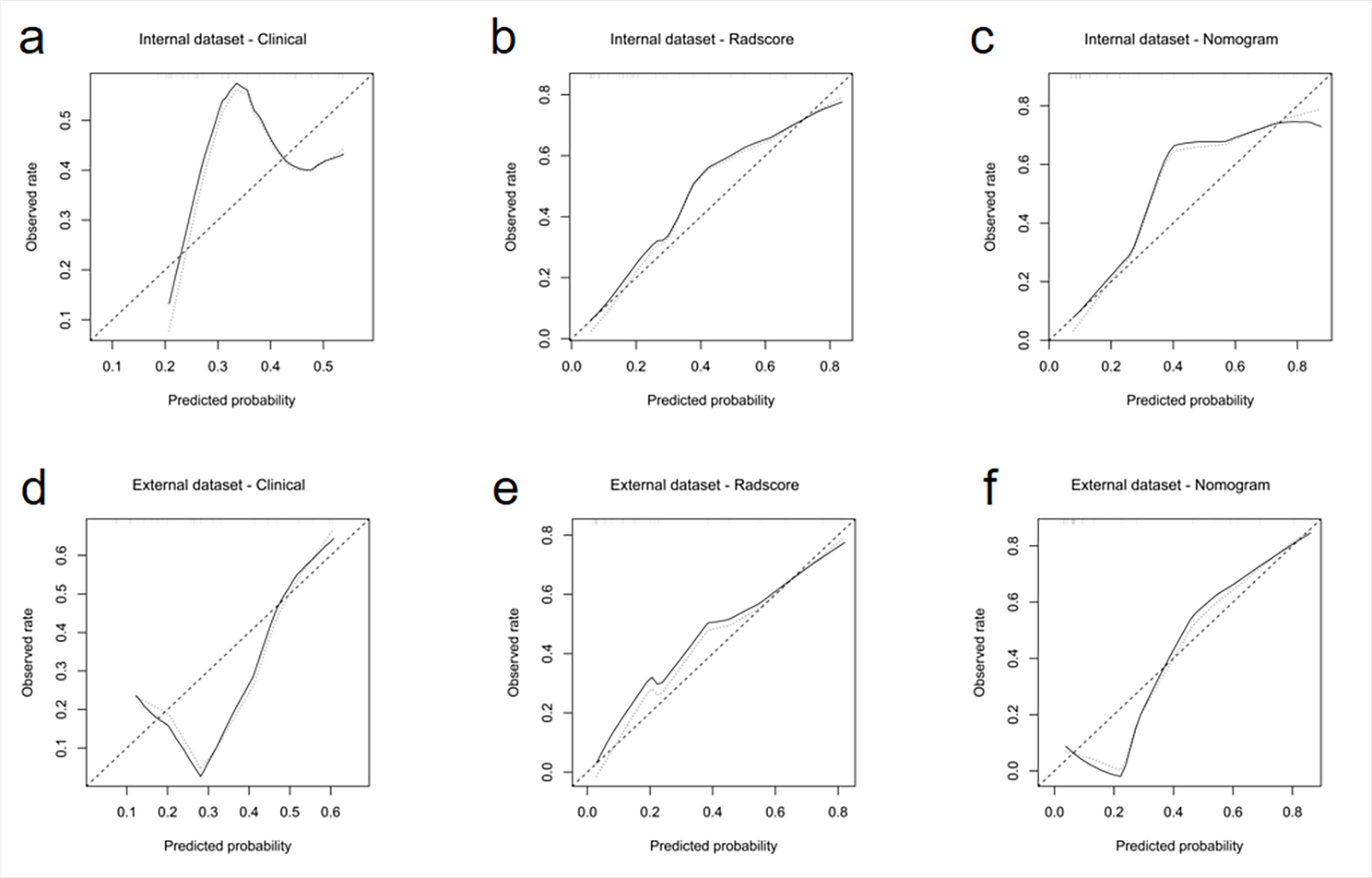


**Fig. S5** Calibration curves of the clinical model, Risk-score, and nomogram in the internal validation dataset (a, b, and c) and the external validation dataset (d, e, and f). The 45-degree dotted line represents a perfect correlation between the predicted probabilities and the actual observed rate.

**
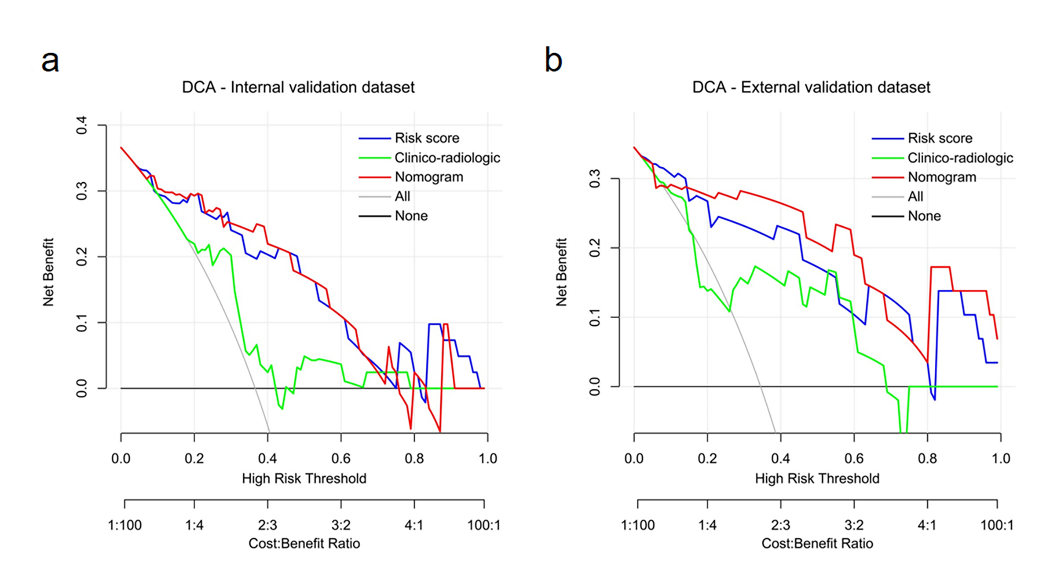
**

**Fig. S6** Decision curve analysis of the clinical model, Risk-score, and nomogram in internal validation dataset (a) and external validation dataset (b).

Notes: DCA, decision curve analysis.
